# A Computer Vision-Based Automatic System for Egg Grading and Defect Detection

**DOI:** 10.3390/ani13142354

**Published:** 2023-07-19

**Authors:** Xiao Yang, Ramesh Bahadur Bist, Sachin Subedi, Lilong Chai

**Affiliations:** Department of Poultry Science, University of Georgia, Athens, GA 30602, USA

**Keywords:** laying hen production, egg quality, defect detection, egg weight, deep learning

## Abstract

**Simple Summary:**

Egg defects such as cracks, dirty spots on the eggshell, and blood spots inside the egg can decrease the quality and market value of table eggs. To address this issue, an automatic method based on computer vision technology was developed for grading eggs and determining defects in a cage-free facility. A two-stage model was developed based on RTMDet and random forest networks for predicting egg category and weight in this study. Results show that the best classification accuracy reached 94–96%.

**Abstract:**

Defective eggs diminish the value of laying hen production, particularly in cage-free systems with a higher incidence of floor eggs. To enhance quality, machine vision and image processing have facilitated the development of automated grading and defect detection systems. Additionally, egg measurement systems utilize weight-sorting for optimal market value. However, few studies have integrated deep learning and machine vision techniques for combined egg classification and weighting. To address this gap, a two-stage model was developed based on real-time multitask detection (RTMDet) and random forest networks to predict egg category and weight. The model uses convolutional neural network (CNN) and regression techniques were used to perform joint egg classification and weighing. RTMDet was used to sort and extract egg features for classification, and a Random Forest algorithm was used to predict egg weight based on the extracted features (major axis and minor axis). The results of the study showed that the best achieved accuracy was 94.8% and best R2 was 96.0%. In addition, the model can be used to automatically exclude non-standard-size eggs and eggs with exterior issues (e.g., calcium deposit, stains, and cracks). This detector is among the first models that perform the joint function of egg-sorting and weighing eggs, and is capable of classifying them into five categories (intact, crack, bloody, floor, and non-standard) and measuring them up to jumbo size. By implementing the findings of this study, the poultry industry can reduce costs and increase productivity, ultimately leading to better-quality products for consumers.

## 1. Introduction

Eggs are a nutritious food source for humans and are widely consumed across the world, but their high fragility and specific appearance make them vulnerable to defects during production [1]. Defects such as cracks, dirty spots on the eggshell, and blood spots inside the egg can decrease the quality and market value of eggs. To address this issue, researchers have developed automatic methods for grading eggs and determining defects. In the past, machine vision and image-processing technology have been applied to egg-quality detection and grading in the USA and abroad. Researchers have built gray-machine-vision systems and trained neural networks using egg image histograms to classify eggs into cracked and grade A [2,3]. They have also established conventional neural networks (CNN) for the detection of blood spots, cracks, and dirt stains and developed an expert system for egg-sorting based on these networks [4,5]. The average accuracy of these systems exceeds the USDA requirements [6]. Therefore, the use of computer vison to grade eggs automatically has the potential to improve the potential efficiency and quality of the egg production process, leading to higher-quality eggs for consumers and increased market value for producers.

Egg weight is another important aspect of egg quality associated with the egg grade and market value [7]. The manual measurement of eggs at the digital scale is a time-consuming and tedious process. To improve the efficiency of the egg weighting process, automated egg measurement systems have been developed. Payam et al. (2011) used the ANFIS model to predict egg weight according to the number of pixels of eggs reaching 0.98 R-squared (R^2^) [8], which is more efficient and accurate compared to manual methods. Jeerapa et al. (2017), using the Support Vector Machine (SVM) technique to predict brown chicken eggs from a single egg image, yielded the correlation coefficient of 0.99 [9]. Raoufat et al. (2010) built a computer vison system to measure egg weights by artificial neural networks (ANN); their algorithms showed a high accuracy (R^2^ = 0.96) [10].

Previous works in this area primarily focused on using computer vision techniques such as convolutional neural networks (CNNs) and image classification algorithms for egg classification [11,12]. These methods have shown promising results in classifying eggs based on their size, shape, and color. However, few studies have combined deep learning and machine learning regression techniques for joint egg classification and weighting, especially including floor eggs collected from cage-free poultry farms, which is an important category for real-world egg types, which range from floor eggs to commercial eggs. This can be useful for producers who want to ensure consistent quality across all types of eggs and consumers who want to purchase high-quality eggs. Another reason for this is that the egg industry is shifting from cage to cage-free [13,14,15,16]. Therefore, introducing floor eggs is beneficial for application in the cage-free egg industry.

In this study, an automatic system will be developed at the University of Georgia, aiming to fill this gap by integrating deep learning and supervised machine learning technologies to perform joint egg classification and weighting. The system uses an updated and powerful CNN, called real-time multitask detection (RTMDet), to extract egg features for classification [17], and a classic Random Forest (RF) algorithm to regress egg-weight data based on the extracted features [18]. The objects of this study were as follows: (1) develop an egg classifier to sort eggs through their size and surface; (2) build a regressor to predict egg weights through their geometrical attributes; (3) combine egg-sorting and the measuring of egg weights into one two-stage model; (4) test the model with standard eggs and second eggs. This two-stage model is expected to result in improved accuracy and efficiency compared to existing methods.

## 2. Materials and Methods

### 2.1. Egg Collection

**Figure 1:**
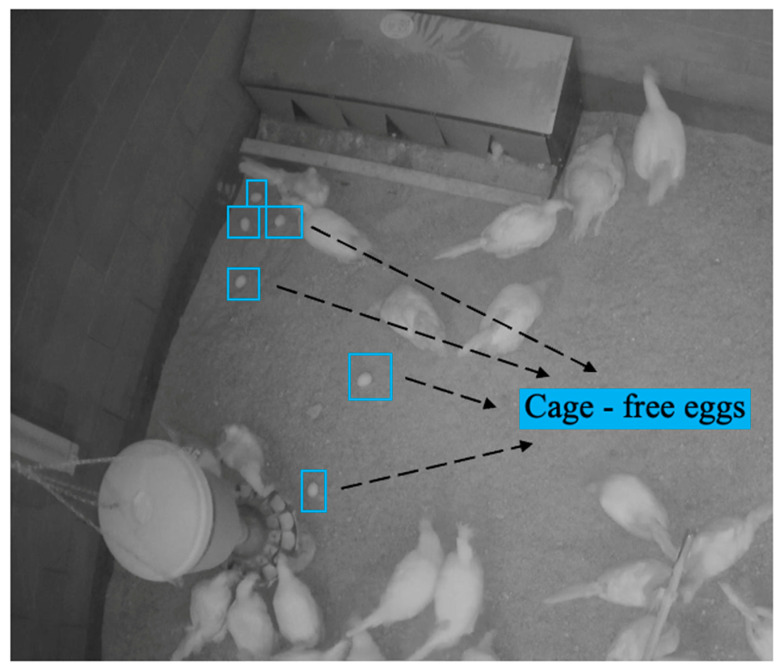
The production of cage-free eggs.

**Figure 2:**
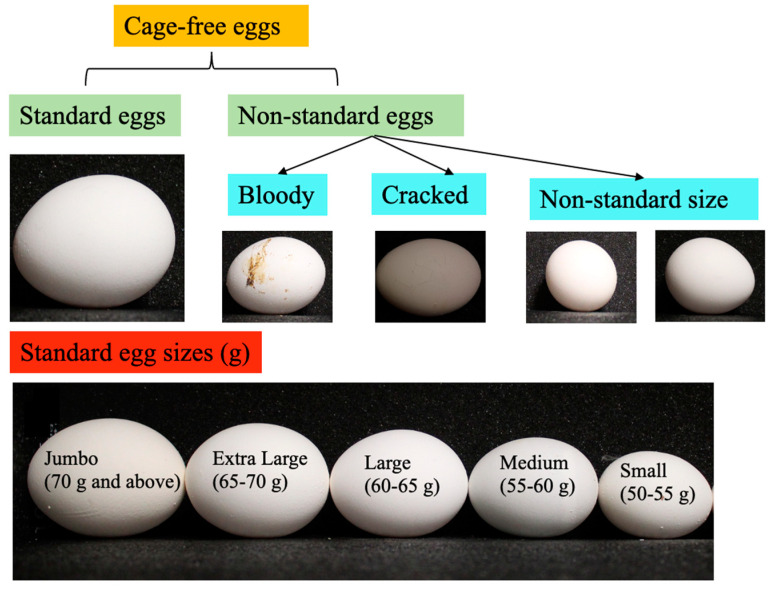
The classification of cage-free eggs and visualization of standard egg sizes (g).

In this study, 800 Hy-line W-36 hens were used to produce cage-free eggs with free access to fresh water and feeds (Figure 1). The eggs were collected daily and stored at a temperature around 24 °C for the next sorting process, and were then graded according to size and quality. A binary classification (standard and defect eggs) was first introduced to classify the eggs manually. The standard eggs were those that were clean, and sizes ranged from small (50–55 g) to jumbo (70 g and above), while the non-standard eggs were those that were bloody, cracked, had an unusual egg shape (too long, too round or distorted), and a size less than small or more than jumbo (Figure 2) [9,19]. This classification was applied to determine the quality of the eggs and to ensure that only the best quality eggs were utilized for measuring egg weight.

### 2.2. Egg Samples Acquisition System

**Figure 3:**
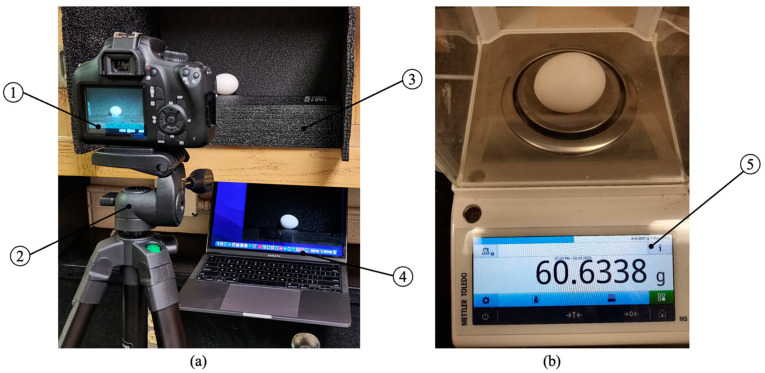
The egg samples’ acquisition system for classifying eggs (**a**) and weighting eggs (**b**): (1) camera; (2) tripod; (3) egg base; (4) computer; (5) digital scale.

An egg samples’ collection system was constructed to collect images and weights of different classes of eggs at the department of poultry science at the University of Georgia (UGA), USA. Figure 3 demonstrates the egg sample acquisition setup, including the camera, tripod, egg base, computer, and digital scale. Details are shown in Table 1. The system is designed to accurately collect and record data on the different classes of eggs. The camera, which is mounted on a tripod, takes images of the eggs placed on the designated egg base. The digital scale measures the weight of the eggs, and the computer stores the collected data and images. The combination of the camera, scale, and computer allows for a comprehensive and efficient egg sample collection process. The collected data and images were used to develop an automatic system for classifying and weighting the eggs using computer vision.

### 2.3. Egg Data Processing

**Figure 4:**
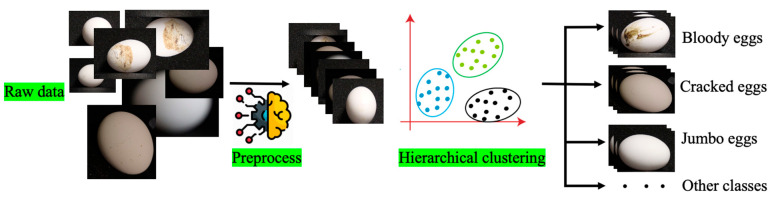
The flow of egg data processing.

Once the egg image data have been collected, they undergo two key processing steps: preprocessing the diffraction patterns and performing hierarchical clustering on the data. These steps involve refining the diffraction patterns and organizing the data into clusters based on their similarities [20]. Preprocessing involves removing background noise, normalizing the signal intensity, and correcting for any artifacts in the data. This step ensures that the diffraction patterns are clean and reliable for analysis. Hierarchical clustering is a method for grouping similar data points into clusters based on their similarity [21]. The algorithm starts by considering each data point as its own cluster, and then iteratively merges clusters until a desired number of clusters is reached or a stopping criterion is met (Figure 4). This approach can be used to identify patterns in the egg data, such as different eggshell types (bloody, cracked and distorted) or quality grades (small size to jumbo size).

### 2.4. Egg Sorting Method

To develop a real-time automatic egg-quality checking system that meets the future requirements of the egg industry by utilizing deep learning for small object classification, specifically egg classification, during the grading process, a new family of original real-time models using you only look once (YOLO) for object classification, referred to as RTMDet, was utilized. RTMDet is introduced with improved small-object detection abilities. The appealing enhancements come from the large-kernel depth-wise convolutions and soft labels in the dynamic label assignments. This approach enables a comprehensive egg analysis, encompassing factors such as egg size and eggshell type. The large-kernel depth-wise convolutions improve the model’s global context-capturing ability, while reducing the model depth to maintain a fast inference speed. The training strategies are revisited to improve accuracy with a better combination of data augmentations and optimization. Soft targets are introduced instead of hard labels in the dynamic label assignment process, improving discrimination and reducing noise in label assignment.

**Figure 5:**
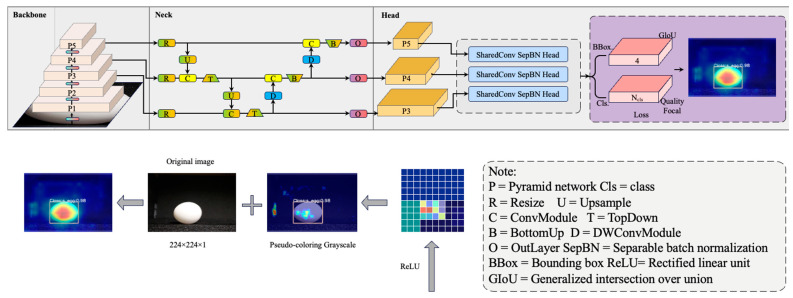
The structure of egg classification based on RTMDet architecture.

The overall architecture of the RTMDet classifier is broken down into three parts: the backbone, neck, and head. The backbone component is similar to that of YOLO, which is a recent advance in object detection, and is regularly equipped with a cross-stage partial network darknet (CSPDarkNet). This backbone consists of four stages, each of which is composed of several basic neural layers. These layers are designed to extract hierarchical features from the input data, capturing both low-level and high-level visual information. The neck merges the multi-scale feature pyramid from the backbone and improves it through bottom-up and top-down feature flow. It facilitates the fusion of information across different scales, enabling the model to effectively handle objects of various sizes. This ability is especially relevant when considering parameters such as the major axis and minor axis of the eggs. The major axis corresponds to the longer diagonal of the egg, providing insights into its overall length and shape. On the other hand, the minor axis represents the shorter diagonal, which helps to assess the width of the eggs. The detection head then identifies the object bounding boxes and categorizes them using the feature map at each scale. By analyzing the feature maps at different scales, the detection head can accurately localize objects and assign corresponding class labels (standard, bloody, floor, cracked and non-standard). This design is well-suited to both standard and small objects and can be expanded to instance segmentation through the implementation of kernel and mask feature production modules [17]. To provide a clearer representation of the system architecture, a diagram of the RTMDet macro-architecture is shown in Figure 5 [17].

#### 2.4.1. Large-Kernel Depth-Wide Convolution Approach

Large-kernel depth-wise convolutions involve the use of more extensive filters in depth-wise convolutional layers within a convolutional neural network (CNN) [22]. The purpose of using these larger kernels is to gain a better understanding of the contextual information contained in the input data and enhance the representation power of the model. Depth-wise convolutions are frequently utilized in CNNs to reduce computational complexity and boost efficiency. Nevertheless, they have limitations in capturing significant scale context and spatial information. With the use of large-kernel depth-wise convolutions, this constraint can be overcome. The advantages of using large-kernel depth-wise convolutions include improved model ability when applied to real-world objects, a more comprehensive capturing of the data and their surroundings, and enhanced accuracy on benchmark datasets. In the context of egg classification, this approach allows for a more comprehensive analysis of various parameters, including egg size, eggshell type, and other spatial characteristics. Furthermore, large-kernel depth-wise convolutions allow for a reduction in the number of parameters and computation, while still delivering a similar performance to models with more parameters.

#### 2.4.2. Soft Labels

In deep learning, soft labels refer to the use of continuous, rather than binary, values as target outputs. The purpose of using soft labels is to provide the model with additional information and to encourage smoothness in the model predictions [19,23]. By employing soft labels, the model can generate predictions that provide more subtlety and precision in the classification task. Instead of solely assigning eggs to specific classes with binary labels, the soft labels enable the model to express varying degrees of confidence or probabilities for each class. This allows for a more detailed understanding of the eggs’ characteristics and their association with different classes. In addition, the use of soft labels can result in more robust models because the model is able to discover correlations between the input data and the desired outputs, even if the relationship is not obvious. In our study, soft labels are applied in problems with multi-class classification or multi-label classification (i.e., unclean eggs, standard eggs, and no standard eggs), where the model must predict the presence of multiple target classes [24,25]. In addition, on the basis of simplified optimal transport assignment (SimOTA), an advanced cost function calculation for soft labels was presented to reduce training loss, and its loss function is described below.

(1) $$\;f\left(C\right)=\alpha_{1}{f(C}_{cls})+\alpha_{2}\;f\left(C_{reg}\right)$$

where $f\left(C\right)$ is loss fuction, ${f(C}_{cls})$ is the classification loss,$\;{f(C}_{reg})$ is the regression loss, and two coefficients, $\alpha_{1}\mathrm{and}\alpha_{2}$, were empirically set.

(2) $${\;\;f(C}_{cls})=CE\left(P,Y_{soft}\right)\times {\left(Y_{soft}-p\right)}^{2}\;\;$$

where $CE\left(P,Y_{soft}\right)$ represents the cross-entropy (*CE*) loss between the predicted probabilities (*P*) and the soft labels ($Y_{soft})$.

(3) $$f\left(C_{reg}\right)=-\log\left(IoU\right)$$

where −log (*IoU*) means the negative logarithm of the intersection over union (*IoU*).

### 2.5. Egg Weight Prediction Method

**Figure 6:**
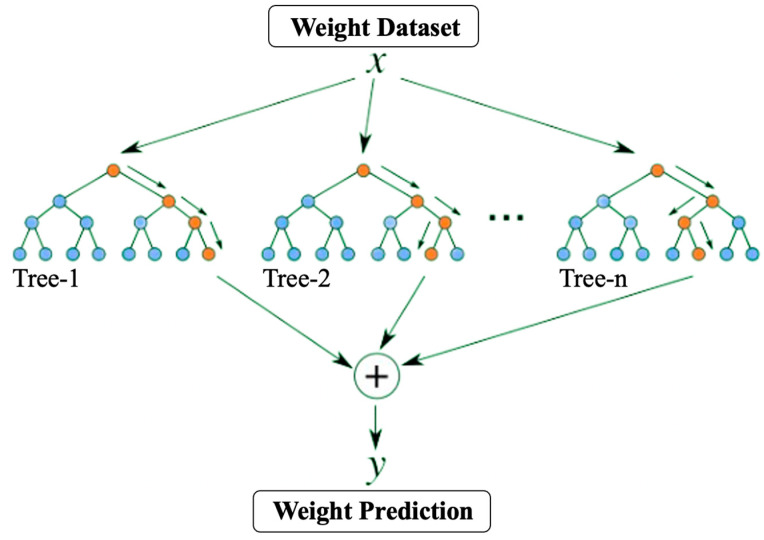
Random forest algorithm.

Predicting egg weight through computer vision leads to several challenges that must be addressed. One of the challenges is the accuracy of measurements of the egg’s dimensions, such as the major and minor axis. This is due to the difficulty of obtaining high-quality images or accurately identifying and measuring the egg in the image. Another obstacle is the diversity in the shapes and sizes of eggs (small–jumbo), which requires the implementation of complex machine learning algorithms that can account for various factors, including eggshell color, shape, size, and birth date, that may affect egg weight. Random Forest Regression is utilized for egg-weight prediction due to its ability to handle complex, non-linear relationships between features and target variables using an ensemble learning method that combines predictions from multiple decision trees, which are trained on randomly selected subsets of the data. This combination reduces variance and enhances the overall accuracy of the model. Furthermore, Random Forest can handle missing or incomplete data and perform effectively when there is a combination of continuous and categorical variables [18,26]. Lastly, feature importance scores are provided by Random Forest, which helps determine the most significant factors that contribute to egg weight prediction. The structure of RF is shown below (Figure 6) [27].

### 2.6. Computer Vision System

**Figure 7:**
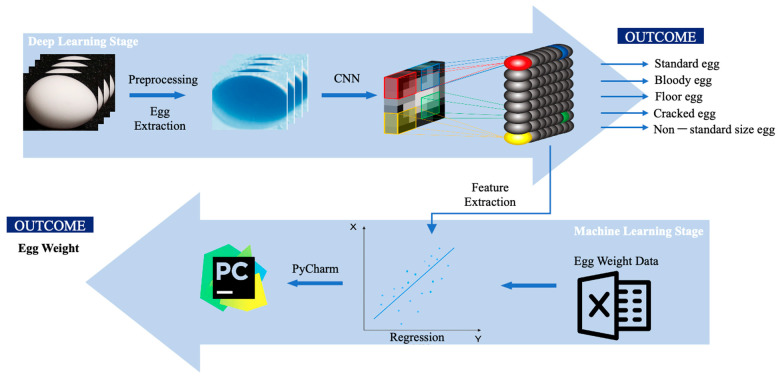
A streamlined approach to egg quality classification using computer vision.

**Figure 8:**
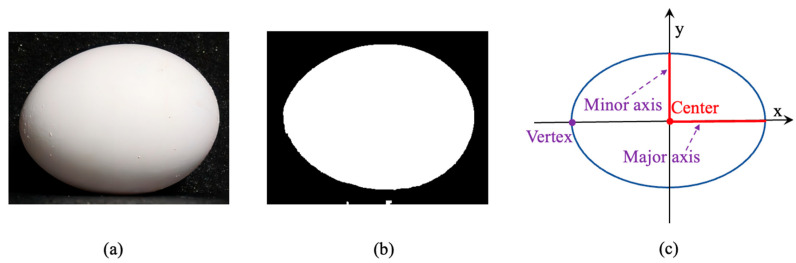
The processes of calculating egg parameters: (**a**) original image; (**b**) binary image; (**c**) geometric image.

In this study, we aim to integrate computer vision technologies, deep learning and machine learning, into a single implementation for the purpose of jointly performing egg-sorting and weighting functions. The input egg images will first be processed through RTMDet, a deep learning technique that surpasses conventional CNN models, to extract egg features for classification. After obtaining the segmented mask of the egg, we identify four cutting points on the mask, namely, the top, bottom, left, and right points. These points are then used to form a new rectangle. Within this rectangle, the longer diagonal corresponds to the major axis, while the shorter diagonal corresponds to the minor axis. The weighting function will then utilize a classic Random Forest algorithm to regress egg weight data based on the egg features (major axis and minor axis) extracted by binary image. Figure 7 and Figure 8 show the whole flow [28].

### 2.7. Performance Evaluation

In this research, a dataset was created using 2100 egg images, which were then randomly divided into training and testing sets with a ratio of 4:1. To better analyze and compare performance across egg classes, the confusion matrix was created to derive standard parameters in classification tasks [29]. The confusion matrix is a two-dimensional table that summarizes RTMDet model’s performance by comparing the predicted and actual class labels. Each row of the matrix represents occurrences in a predicted class, while each column represents instances in an actual class. The elements of the confusion matrix represent the number of cases identified correctly versus incorrectly. The four elements of true positives (*TP*), false positives (*FP*), true negatives (*TN*), and false negatives (*FN*) are used to calculate evaluation metrics such as precision, recall, F1-score, and average precision (*AP*) for egg grading in deep learning [30,31]. To further explore the performance of Random Forest, coefficient of determination (*R*^2^) is utilized to evaluate the goodness of fit of the regression model.

(4) $$precision=\frac{TP}{TP+FP}$$

(5) $$recall=\frac{TP}{TP+FN}$$

(6) $$F1-score=\frac{2\times \left(precision\times recall\right)}{\left(precision+recall\right)}$$

(7) $$\;\;AP=\int_{\gamma =0}^{1}p\left(r\right)dr\;\;$$

where *p*(*r*) means the precision–recall curve.

(8) $$R^{2}=1-\frac{{SS}_{res}}{{SS}_{tot}}=1-\frac{\sum {\left(y_{i}-{\hat{y}}_{i}\right)}^{2}}{\sum {\left(y_{i}-\bar{y}\right)}^{2}}$$

where *SS_res_* represents the residual sum of squares and *SS_tot_* means the total sum of squares.

## 3. Results

### 3.1. CNN Model Comparison

**Figure 9:**
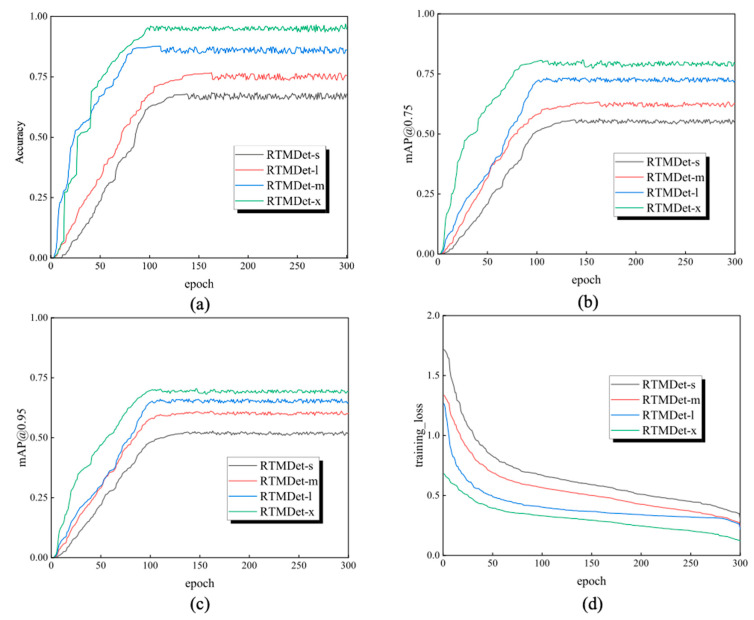
Model comparison: (**a**) accuracy, (**b**) mAP@0.75, (**c**) mAP@0.95 and (**d**) training loss.

Four individual experiments (RTMDet-s, RTMDet-m, RTMDet-l and RTMDet-x) were conducted to discover the optimal classifier for egg-sorting. All experiments trained 300 epochs based on Python 3.7 version, PyTorch deep learning library and a hardware with NVIDIA-SMI (16 GB) graphics card. A summary of the model comparison is listed below (Table 2). In terms of accuracy, RTMDet-x reached an accuracy of 94.80%, which was better than any other comparison model. Correspondingly, the training loss and validation loss values of RTMDet-x were also the smallest among all the tested models because fewer loss values mean minor errors in neural networks. In terms of floating-point operations per second (FLOPS), RTMDet-s with fewer parameters have minimal FLOPS compared with other methods, which means they requires less computational time to perform a forward or backward pass in a neural network, and therefore have a broader further application in robots with limited computational resources [32]. In addition, RTMDet-x also outperformed any other comparison model in map@0.75 and map@0.95 because of the additional parameters required for the computer to perform classification. Figure 9 shows the detailed comparison results of the model indicators for different deep learning classifiers. These findings demonstrated that RTMDet-x achieved the best performance in terms of egg classification.

#### Results of Classification of Egg Sorting

**Figure 10:**
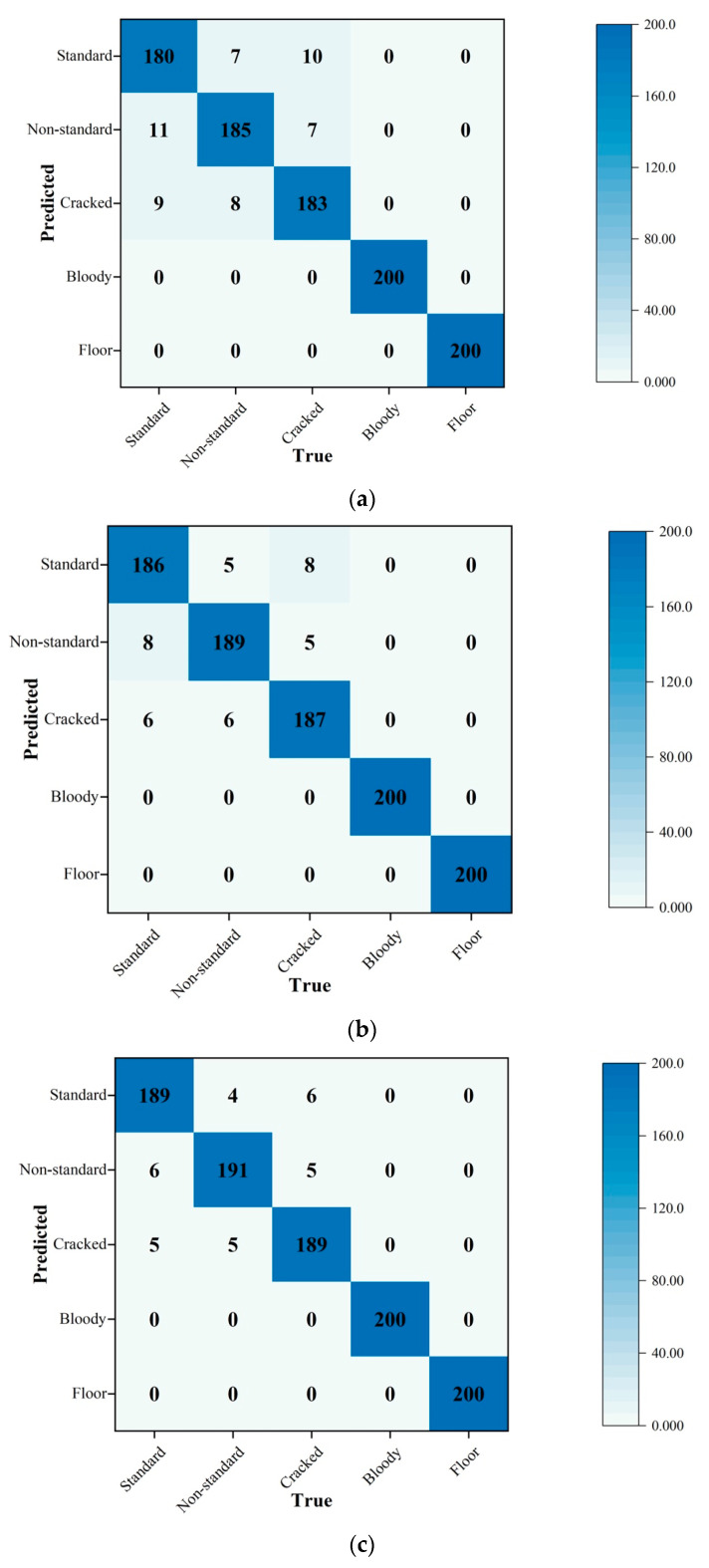

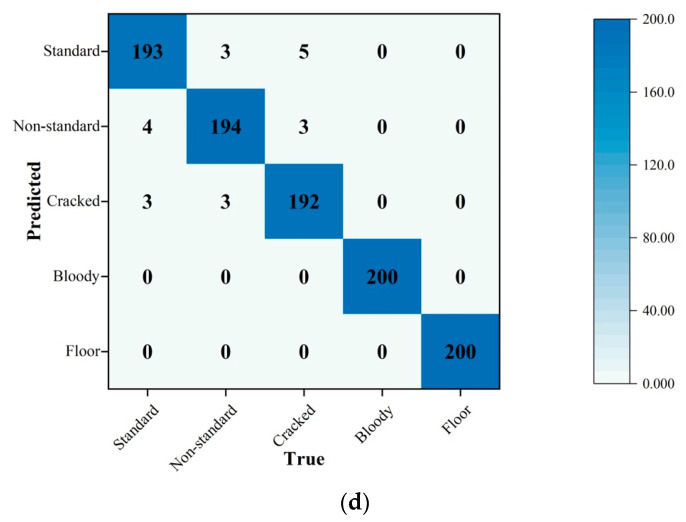
Confusion matrix of classifiers for different types of eggs ((**a**–**d**) represent RTMDet-s, RTMDet-m and RTMDet-l and RTMDet-x, respectivly).

To compare the classification performances of multiple deep learning models on the classification of eggs, the confusion matrix was adopted (Figure 10). Each type of egg was tested by different models 200 times.

**Figure 11:**
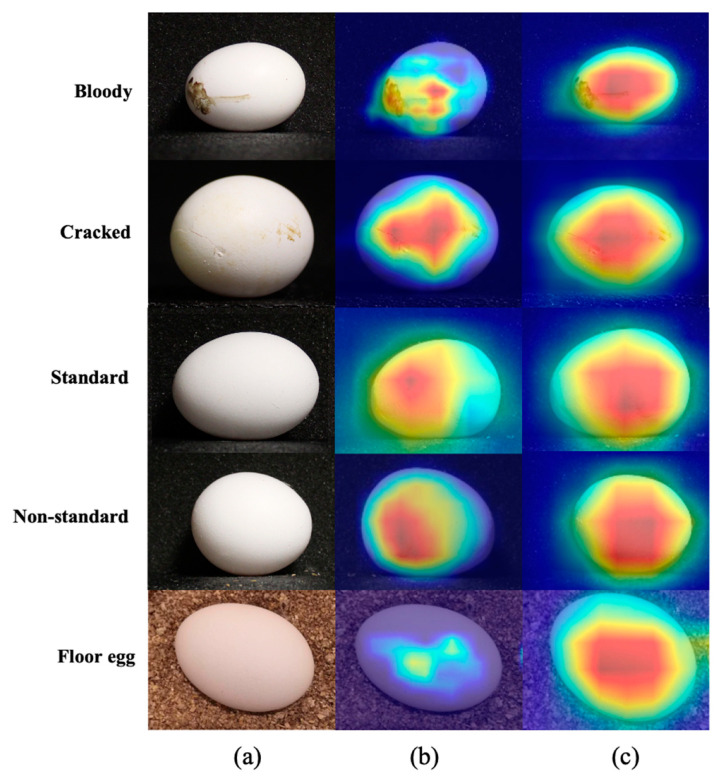
Visualization of CNN: (**a**) original image, (**b**) heatmap and (**c**) gradient-weighted map.

The prediction results are shown in the confusion matrix, where the gradually changing shade of blue represents the accuracy of true predictions (cells filled with deeper blue have more accurate predictions). The number in each cell represents the results of the models [33]. The average true scores (along the diagonal line from the top-left corner of the matrix to the bottom-right corner) of RTMDet-x are the highest among the whole confusion matrix of classifiers, which indicates that RTMDet-x has a better true prediction rate. The scores off the diagonal (false scores) represent the instances where the predicted class does not match the true class. The average false scores of RTMDet-s are higher than those of other classifiers, which means its performance could be improved. In terms of type error, no type error was observed in the classes of bloody eggs and floor eggs. The reason for this is their significant characters; for example, bloody eggs have clear bloody spots and only floor eggs have a litter background. However, when classifiers detect eggsusing standard, non-standard, and cracked eggs, some errors exist due to the similarities within the minor axis and major axis, and the difficulties in detecting microcracks and cracks located on the bottom or sides not shown by the camera [34]. However, the results were still acceptable because there are not many non-standard eggs or cracked eggs on commercial poultry farms (varying between 1 and 5% of the total) [35]. In general, the RTMDet-x classifier is the best experimental classifier with the highest accuracy. In addition, to visualize how RTMDet-x classifies eggs and extracts feature maps, heatmap and gradient-weighted class-activation mappings were outputted (Figure 11). To understand the model’s decision-making process and identify important regions in the input images, the gradient-weighted class activation mapping (Grad-CAM) technique was utilized [36]. Grad-CAM produces a heatmap that highlights the regions contributing significantly to the model’s predictions. By extracting the feature map from the last convolutional layer of the input egg image, a Grad-CAM heatmap is created. The feature map channels are then weighted using a class gradient computed with respect to the feature map. This weighting process emphasizes regions that strongly influence the model’s predictions. Experimental findings demonstrate the CNN-based model’s ability to effectively extract features from areas with blood spots and broken parts, even when the defects are minor. This showcases the model’s capacity to accurately identify egg abnormalities and make precise predictions.

### 3.2. Regression Results

**Figure 12:**
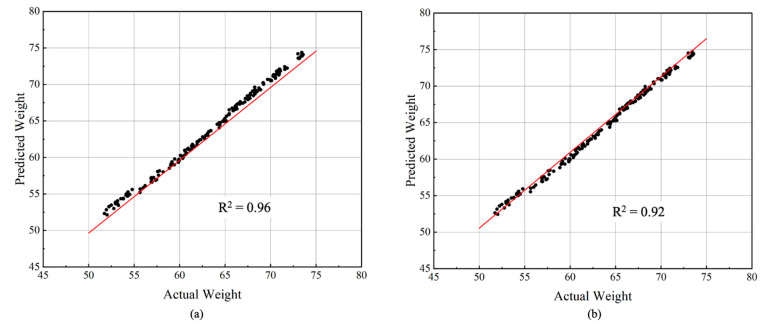
Regression models with (**b**) or without (**a**) storage date factor.

In this study, a random forest (RF) regressor was used to predict standard eggs (from small to jumbo size) because only standard eggs (consistent size and weight) can be sold to consumers by commercial poultry farms. As shown in Figure 12a, the predicted weight, using minor and major axis features using the RF regressor, showed an R^2^ value of 0.96, which suggests that the predicted weights were highly correlated with the actual weights of the eggs. To further analyze the best performance of RF regressor, we classified standard eggs into five types (small, medium, large, extra-large and jumbo) and test each type 100 times using an RF regressor. In addition, the storage of eggs is another important factor affecting egg weight; therefore, we also include this when comparing the predicted weight using minor and major axes obtained using the random forest regressor and the actual weight of the eggs on different storage days (R^2^ = 0.92) (Figure 12b). By comparing the predicted weight obtained using the random forest regressor with the actual weight of the eggs under different storage conditions, the study was able to evaluate the robustness of the regressor in accounting for storage effects. Our storage conditions (24 °C) had a minimal impact on egg diameter, which remains highly correlated with egg weight [37]. As a result, the RF regressor can continue to accurately predict egg weight. The stable storage temperature ensures that the regressor’s accuracy in estimating egg weight remains unaffected. The results suggest that the regressor is able to accurately predict egg weight under different storage conditions, which can be useful for optimizing egg production and storage practices [38].

### 3.3. Results of Weighting Eggs

**Figure 13:**
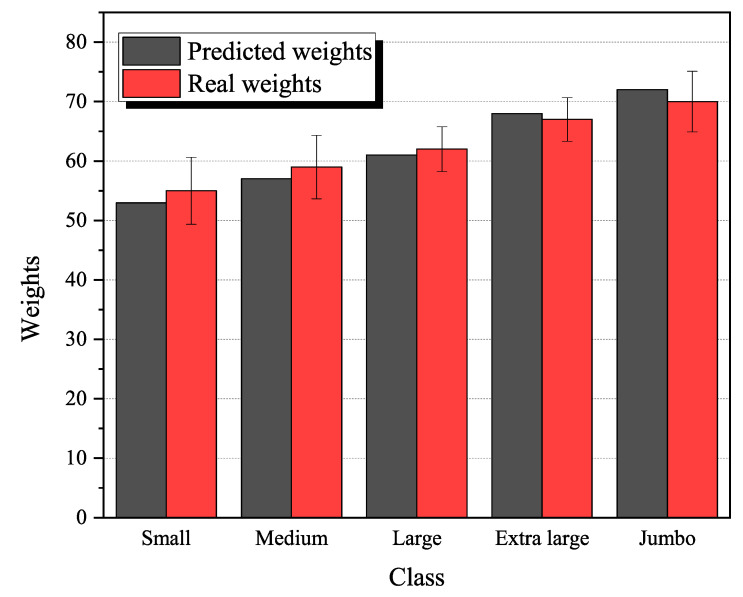
Egg weight prediction from small to jumbo.

To further test the model under egg scales ranging from small to jumbo, each category randomly selected 100 pictures to test the robustness and precision of the regressor. The results are shown in Figure 13. The error bar at the top of each stacked bar graph represents the standard error of each class and the height of the green bar represents the absolute error between real weights and predicted weights. From the graph, we can find the height of the error bar for small, medium and jumbo eggs is lower than that for large and extra-large eggs, which indicates that the regressor has a better prediction performance for large and extra-large eggs. This may because the large and extra-large eggs have medium values according to the regression model; in a large dataset, the relationship between the precited variables and the response variables is more complex, resulting in the risk of overfitting and more prohibitive computational costs. However, the data in the medium values may be less affected by measurement error or other types of noise than very small or very large values [39,40]. This can help to improve the accuracy of the regressor predictions. In addition, for some types of data, preprocessing can be simplified for medium values. For example, scaling or normalization may not be as critical for medium values as it is for very small or very large values. In addition, medium values may be complex enough to require a more sophisticated model, but not so complex that the model becomes difficult to interpret. This can help strike a balance between model performance and interpretability.

## 4. Discussions

### 4.1. Discussion of Egg Classification Accuracy

In this study, five classes of eggs were investigated to build a classifier to sort eggs. For floor and bloody eggs, there is no misunderstanding in the classification of them and other classes. This is due to the clear features of floor and bloody eggs [41]. For floor eggs, the eggs are laid in the litter, so, in computer vision, the white eggs are surrounded by brown litter, which is a unique feature compared to other egg classes. This improves the egg classifier’s accuracy when sort it. As for bloody eggs, because of the red spots that appear on white eggshells, there is a clear indicator that the CNN model can use to extract feature maps, and the egg classifier also has a high sorting accuracy. More false classifications are obtained for standard, non-standard and cracked eggs. This is because the classifier uses minor and major axes to differentiate egg size, and non-standard eggs have more abnormal shapes, such as being too long or too round, which means there might be unusual minor and major axes that the classifier misunderstands [5]. In addition, cracked eggs are also not easy for the classifier to detect. This is due to the limitations of camera angles. In this study, we only use the front view of eggs for egg classification tasks. Therefore, some cracks on the eggshell on the back or side view of might be ignored, and cracked eggs will be classified as other types of eggs.

To further discuss the performance of the classifier, we compare our study with various other pieces of research. Table 3 shows the results of some studies conducted on the classification of eggs using computer vision and compares these with the results obtained in the present study. Pyiyadumkol et al. (2017) developed a sorting system based on the machine vision technique to identify cracks in unwashed eggs [42]. The egg images were captured under atmospheric and vacuum pressure. The cracks were detected using the difference between images taken under atmospheric and vacuum conditions. A combination of machine vision methods and the support vector machine (SVM) classifier was presented in Wu et al. (2017) to detect intact and cracked eggs [43]. Guanjun et al. (2019) introduced a machine vision-based method for cracked egg detection [44]. A negative Laplacian of Gaussian (LoG) operator, hysteresis thresholding method, and a local fitting image index were used to identify crack regions. Amin et al. (2020) proposed a CNN model using hierarchical architecture to classify unwashed egg images based on three classes, namely intact, bloody, and broken [45]. In our study, we introduced more classes, floor and non-standard eggs, to cover all the normal egg categories while maintaining a high level of accuracy through the use of the large-kernel depth-wide convolution approach and soft labels, and cooperation with other optimizations such as anchor-free object detection and deformable convolutional networks, which further improve accuracy and efficiency in multi-classification tasks.

### 4.2. Discussion of Egg Weight Prediction Accuracy

Five different graded eggs were predicted and their average absolute error ranged from 0.9 to 1.8 g. Overall, large and extra-large grades have more accurate prediction than small, medium and jumbo eggs. One reason why a larger egg grade (such as large and extra large) may lead to more accurate predictions than smaller grades (such as small and medium) is that larger eggs generally have a higher mean weight than smaller eggs. This means that there is less variation in egg weight within the larger grades, which can make it easier for the regression model to accurately predict the weight of these eggs. On the other hand, smaller eggs and jumbo grades typically have a wider range of weights, which can make it more difficult for the regression model to accurately predict their weights. Additionally, smaller eggs and jumbo grades may also have more variability in their physical characteristics (such as shell thickness and yolk size), which can further complicate the prediction process.

To further investigate the performance of the regressor, we compared our regressor with other egg weight regressors. Table 4 shows the results of some studies conducted on the regression models.

Cen et al. (2006) developed an egg weight detector by an indicator composed of R, G, B intensity and egg diameters [46]. An equation was created by the regression model, and a 97.8% correlative coefficient was achieved. Similarly, Alikhanow et al. (2015) constructed several equations based on different variables (egg area, egg volume, egg minor axis or major axis) [47]; the most significant parameter was egg area, reaching 94.3% R^2^. Other researchers also used computer vison to predicted egg weight based on the regression model, but they used the multi-flow production line in real-time to cooperate with industrial applications. The identical objects’ measurements under a multi-light source was found to be around 95.0% [48]. In our study, we extended the previous egg weight prediction for the upper litter from extra-large to jumbo, but our regressor maintained a high accuracy with non-line regression because a random forest model is an ensemble of decision trees trained on random subsets of the egg weight data and features (major and minor axis). The random forest model’s final prediction is a weighted average of the egg-weight predictions of the individual trees. Since each decision tree in a random forest can model the non-linear relationships between the input features and the target variable, the random forest model, as a whole, can account for nonlinearities in the egg-weight data.

### 4.3. Discussion of Jointly Performing Egg-Sorting and Weighting Functions

**Figure 14:**
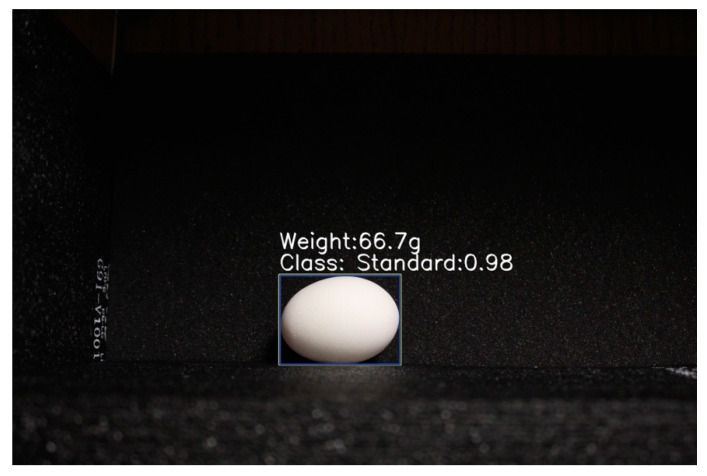
The egg has been classified as ‘Standard’ and its predicted weight is 66.7 g.

In our study, we combine egg classification and weighting tasks into one two-stage model. The approach is to train two distinct models, one for classification and one for regression, and then combine their predictions at the time of inference. First, train a classification model to predict each input’s egg class label. Then, using the predicted class labels to filter the inputs, train a regression model using only the filtered inputs. Use the egg classification model to sort eggs and the corresponding regression model to predict the weight of eggs at same time (Figure 14). The overall performance of the two-stage model is good, but other factors restrict its application, including potential errors in filtering and increased complexity. The classification model is used to filter the regression model’s inputs. If the classification model’s predictions are inaccurate, it may erroneously exclude inputs that the regression model could have used. This can result in a reduction in the accuracy of the final prediction. In addition, the two-stage model approach requires the training of two distinct models and additional processing steps at the time of inference to combine the predictions. This could make the overall architecture more complicated and increase the required computational resources.

### 4.4. Future Studies

Despite the research;s high performance in sorting egg quality based on egg surface and weight, some further studies could the model be applied to real-world situations: (a) using emerging nonvolatile memory (NVM) to reduce memory footprint and latency [49], which is crucial for mobile application; (b) extending the model to egg datasets with more diversity (other egg colors, egg multiplication and other spices) to fulfill the application environment; (c) using a 360-degree camera to prevent misidentification in cracked and bloody eggs; (d) optimize the sorting and weighing process to reduce the time required to complete the task without sacrificing accuracy; (e) enhancing the accuracy of egg segmentation by leveraging the segment-anything model [50].

## 5. Conclusions

In this study, a two-stage model was developed based on RTMDet and random forest networks to predict egg category and weight. The results show that the best classification accuracy was 94.80% and 96.0% for the R^2^ regression model. The model can be installed on the egg-collecting robot to sort eggs in advance and collect our target eggs specifically. In addition, the model can be used to automatically pick out non-standard size eggs and eggs with surface defects (blood-stained or broken). Furthermore, 1000 egg pictures were utilized to test the detector’s performance for different egg types and egg weight scales. The results showed that the detector has a better classification performance for standard and non-standard size eggs, and large (55–60 g) and extra-large (60–65 g) egg weights led to more reliable predictions. This detector is one of the first models that performs the joint function of egg sorting and weighting. By implementing the findings of this study, the poultry industry can reduce costs and increase productivity, ultimately leading to better-quality products for consumers.

## Figures and Tables

**Table 1:** The details of the egg sample acquisition setup.

Parts	Details
Camera	Canon EOS 4000D (Tokyo, Japan)
Tripod	BOSCH BT 150 (Gerlingen, Germany)
Egg base	ESS—8010 (Wasco, CA, USA)
Computer	Apple MacBook Pro (M1, 2020) (Cupertino, CA, USA)
Digital scale	Mettler Toledo MS104TS/00 (Greifensee, Switzerland)

**Table 2:** Summary of model comparison.

Model	Accuracy (%)	mAP@0.75 (%)	mAP@0.95 (%)	Params (M)	FLOPS(G)	Training Loss
RTMDet-s	67.8	55.8	52.3	8.89	14.8	0.30
RTMDet-m	75.6	62.6	60.1	24.71	39.27	0.23
RtMDet-l	86.1	72.1	64.8	52.3	80.23	0.21
RtMDet-x	94.8	79.2	69.1	94.86	141.67	0.12

**Table 3:** Comparison of classification accuracy.

Study	Class					Accuracy (%)
	Intact	Crack	Bloody	Floor	Non-Standard	
Priyadumkol et al. (2017) [42]	✔	✔	-	-	-	94
Wu et al. (2017) [43]	✔	✔	-	-	-	93
Guanjun et al. (2019) [44]	✔	✔	-	-	-	92.5
Amin et al. (2020) [45]	✔	✔	✔	-	-	94.9
Our study	✔	✔	✔	✔	✔	94.8

**Table 4:** Comparison of different regressor accuracies.

Study	Egg Size					R^2^ (%)
	Small	Medium	Large	Extra Large	Jumbo	
Cen et al. (2006) [46]	✔	✔	✔	✔	-	97.8
Alikhanow et al. (2015) [47]	✔	✔	✔	✔	-	94.3
Faith et al. (2023) [48]	✔	✔	✔	✔	-	95.0
Our study	✔	✔	✔	✔	✔	96.0

## Data Availability

Data will be available per reasonable request.
